# High Prevalence and Varied Distribution of Antibiotic-Resistant Bacteria in the Rhizosphere and Rhizoplane of *Citrus medica*

**DOI:** 10.3390/microorganisms10091708

**Published:** 2022-08-25

**Authors:** Fang Yang, Yu Wang, Qianwen Liu, Bo Xu, Huan Chen, Yaomen Li, Kun Wang, Guixin Liang, Ruiqi Zhang, Xin’an Jiao, Yunzeng Zhang

**Affiliations:** 1 Jiangsu Co-Innovation Center for Prevention and Control of Important Animal Infectious Diseases and Zoonoses, Yangzhou University, Yangzhou 225009, China; 2 Jiangsu Key Laboratory of Zoonosis, Yangzhou University, Yangzhou 225009, China; 3 Joint International Research Laboratory of Agriculture and Agri-Product Safety of the Ministry of Education, Yangzhou University, Yangzhou 225009, China; 4 Key Laboratory of Prevention and Control of Biological Hazard Factors (Animal Origin) for Agrifood Safety and Quality, Ministry of Agriculture of China, Yangzhou University, Yangzhou 225009, China

**Keywords:** antibiotic-resistant bacteria, rhizosphere, rhizoplane, *Citrus medica*

## Abstract

The plant-associated bacteria, including that in the rhizosphere and rhizoplane, play important roles in human exposure to antibiotic-resistant bacteria (ARB). The rhizosphere and rhizoplane represent two distinct environments with different selective pressures for bacterial colonization. However, whether the difference in characteristics between the rhizosphere and rhizoplane can affect the abundance and antibiotic resistance profiles of ARB colonizing, the two environments remain largely unknown. In this study, we obtained 174 bacterial isolates from the rhizosphere (113 isolates) and rhizoplane (61 isolates) of *Citrus medica* trees grown in a park, where humans could easily and frequently contact the trees. A very high proportion of isolates exhibited resistance to several clinically important antibiotics, including β-lactam class antibiotics and polymyxin, with several known antibiotic-resistant opportunistic pathogens, such as *Micrococcus luteus*, being identified. The prevalence of ARB in the rhizoplane was higher than that in the rhizosphere. While the prevalence of polymyxin-resistant isolates was higher in the rhizoplane, the prevalence of amphenicol-resistant isolates was significantly higher in the rhizosphere. In summary, our findings suggest that the rhizosphere and rhizoplane are important media for the spread of ARB, and the different characteristics between the two environments can affect the distribution of ARB.

## 1. Introduction

Antibiotics are important for treating bacterial infections in humans and animals. They also have nonmedical applications. However, recently, excessive and inappropriate use of antibiotics, which is estimated to be as high as 100–200 thousand tons annually worldwide, has resulted in a substantial increase in the rates of environmental release of antibiotics [1]. The released antibiotics can enter various environmental compartments through several ways, including urban wastewater and agricultural practices, such as the use of antibiotic-polluted manure, among others [1,2]. Such antibiotic release accelerates the spread and evolution of antibiotic-resistant bacteria (ARB) in the environment. Certain ARB, particularly pathogenic ones, may enter the human intestinal tract through direct contact, plant-mediated transmission, and other modes of transmission, thereby seriously threatening public health [3,4,5].

Soil is an important environmental component that receives released antibiotics as well as ARB from wastewater, livestock manure, and several other media. Notably, antibiotics and ARB in the soil get enriched in plant root-associated environments, enter the plants, and then get transmitted to humans via the food chain and direct contact, among others [6,7,8]. For instance, various opportunistic pathogens, particularly ARB, which can infect humans, have been identified in plant root-associated environments, including the rhizosphere and rhizoplane [9,10]. Several taxa belonging to *Enterobacteriaceae*, including several known opportunistic pathogens causing serious foodborne human diseases associated with the consumption of *Eruca vesicaria* (arugula, a salad plant), were found to be key members of the arugula microbiota and were resistant to several antibiotics [11,12]. Thus, the plant microbiome has been recognized be crucial for human exposure to ARB [5].

The rhizosphere and rhizoplane are important environments for microbial colonization and transmission within the plant microbiome [13]. Compared with the rhizosphere, the rhizoplane has more abundant and easy-to-use nutrients but many more severe plant- and microbe-derived stresses for bacterial colonization [13]. Given these distinct differences between the rhizosphere and rhizoplane, the composition of bacterial communities colonizing the rhizosphere and rhizoplane of several plant species is dramatically different. The prevalence of several taxa significantly differs between the rhizosphere and rhizoplane, reflecting the differences in nutrient utilization and stress adaptation strategies of these bacteria in the two environments [13,14,15]. However, whether and to what extent nutrient availability and stress pressure differences between the rhizosphere and rhizoplane can affect the antibiotic resistance profiles of the bacteria colonizing the two environments remain unclear. In this study, we assessed the antibiotic resistance profiles of bacterial strains isolated from the rhizosphere and rhizoplane of *C. medica* at Yangzhou Zhuyu Bay Scenic Spot (Yangzhou Zoo), where humans can closely contact the trees. Modified medium, prepared by autoclaving the phosphate buffer and agar components separately (termed as PS medium) before solidification, was used in this study [16]. A previous study reported that phosphate buffer and agar can react and generate hydrogen peroxide (H_2_O_2_) in the resulting medium (PT medium) when mixed together before autoclave, thereby markedly reducing the cultivation efficiency. By contrast, PS medium can minimize the production of reactive oxygen species (ROS) and adverse compounds, such as H_2_O_2_, which inhibit the formation of microbial colonies [16]. Thus, the cultivability of microorganisms, particularly slow-growing bacteria, can be markedly improved by using PS medium [17]. In this study, we used PS medium to obtain 174 bacterial isolates from the rhizosphere and rhizoplane of *C. medica* (113 from the rhizosphere and 61 from the rhizoplane). We assessed the antibiotic resistance profiles of these isolates using 13 widely used antibiotics to determine the risk of transmission, of ARB to humans, from such easily accessible environments and to understand whether, and to what extent, the distinct characteristics of the rhizosphere and rhizoplane can affect the antibiotic resistance profiles of the bacteria colonizing these environments.

## 2. Materials and Methods

### 2.1. Sample Collection and Bacterial Isolation

Soil samples were collected from the rhizosphere and rhizoplane of four *C. medica* plants grown at Yangzhou Zhuyu Bay Scenic Spot (Yangzhou Zoo) (32.24° N, 119.26° E), Yangzhou, China, on 22 August 2020. The park is located in the northwest of Yangzhou City, with a distance of 5 km from the downtown area, and covers an area of around 50 hectares. Approximately 600–800 guests visit the park each day. The *C. medica* trees were around 15 years old, and they were located in the center region of the park, with rest chairs located 1 m away from the trunk of these trees. The trees were treated with livestock manure at least once every year. The four trees were around 4 m away from each other. The soil samples were collected from four corners around 1 m away from the trunk (Figure S1). Soil cores with fine roots (i.e., roots with approximately 1-cm-thick adjoining soil layers) were collected from the four corners of each tree (more than 1 kg), were mixed together, and placed in sterile ziplock bags. The samples were then placed on ice and quickly brought back to the laboratory for processing. Loosely attached soil on the roots was removed with gentle shaking, and tightly attached soil on the roots (1–2 mm thick) was collected using sterile soft brush pencils. For each of the obtained soil samples from the rhizosphere, 1 g of soil was suspended in 49 mL sterile 0.9% NaCl. Then, the treated fine roots were placed in a 50 mL centrifuge tube containing precooled 0.9% NaCl solution and four to five glass balls (diameter of approximately 5 mm). The samples were vortexed at maximal speed (3200 rpm) for 10 min, following which the fine roots in the centrifuge tube were removed and discarded using a sterile tweezer. The tube containing only the rhizoplane soil suspension was centrifuged at 4 °C at 12,000× *g* for 3 min, following which the supernatant was discarded and resuspended in 49 mL 0.9% NaCl. 

The soil samples from the rhizosphere and rhizoplane were 10-fold diluted to 10^−6^, using 0.9% NaCl solution, and shaken at 200 rpm at 28 °C for 1 h. Following this, 100 µL suspension, from 10^−4^ to 10^−6^, was spread and plated on PS medium [16], with three replications being performed per dilution. The plates were incubated at 28 °C, for 3–10 days, in a bacteriological incubator. Distinct colonies were isolated, purified, and sub-cultured more than thrice in the PS medium.

### 2.2. DNA Extraction and Taxonomic Classification

The DNA of the isolates was extracted using the TIANamp Bacteria DNA Kit (TIANGEN, Beijing, China). The 16S rRNA gene universal primers 27F (5′-AGAGTTTGATCCTGGCTCAG-3′) and 1492R (5′-TACGGCTACCTTGTTACGACTT-3′) were used for PCR amplification. The PCR amplified products were purified and sequenced by Suzhou Jinweizi Biotechnology Co., Ltd., Suzhou, China. The taxonomic affiliation of the isolates was then determined using the EzBioCloud database [18]. The maximum likelihood phylogenetic tree was reconstructed using IQ-TREE [19]. The tree was annotated and visualized using the iTOL online browser (https://itol.embl.de/) (accessed on 17 August 2021) [20]. The 16S rDNA sequences have been deposited in the NCBI GenBank database under accession numbers OP108637 to OP108810.

### 2.3. Antibiotic Susceptibility Assay

The minimal inhibitory concentration (MIC) of 13 widely used antibiotics, namely β-lactam class antibiotics (ampicillin, clofazimine, and cefotaxime), tetracycline, polymyxin, carbapenem class antibiotics (meropenem), aminoglycoside class antibiotics (gentamicin, amikacin, and streptomycin), phenylpropanol class antibiotics (florfenicol), quinolone class antibiotics (nalidixic acid and ciprofioxacin), and chloramphenicol, against the isolates was determined using the agar dilution method [21]. The antibiotics were diluted to approximate concentrations [22], using sterilized MH agar (Mueller–Hinton Broth) solution, at a ratio of 1:19, to prepare antibiotic-containing agar plates. The MH medium (1 L) is comprised of beef extract (3.0 g), acid hydrolysate of casein (17.5 g), starch (1.5 g), and agar (15 g). The isolates were diluted to proper concentrations using liquid PS medium, and the diluents of different isolates were inoculated on the antibiotic-containing agar plates using a multipoint inoculator, followed by incubation at 28 °C for 2–5 days. The MIC of the antibiotics, to inhibit the growth of the isolates, was determined based on the resistance index, as described by the European Committee on Antimicrobial Susceptibility Testing (EUCAST) [22] for each antibiotic, and the resistant or sensitive phenotypes of the isolates were recorded accordingly.

### 2.4. Statistics Analysis

Fisher’s exact test was used to compare the taxonomic affiliation and antibiotic resistance profile differences between the bacterial isolates from the rhizosphere and rhizoplane. The figures were drawn using GraphPad Prism (version 7.0, GraphPad Software Inc.; San Diego, CA, USA).

## 3. Results

### 3.1. Isolation and Identification of Bacterial Isolates from the Rhizosphere and Rhizoplane of C. medica

**Figure 1:**
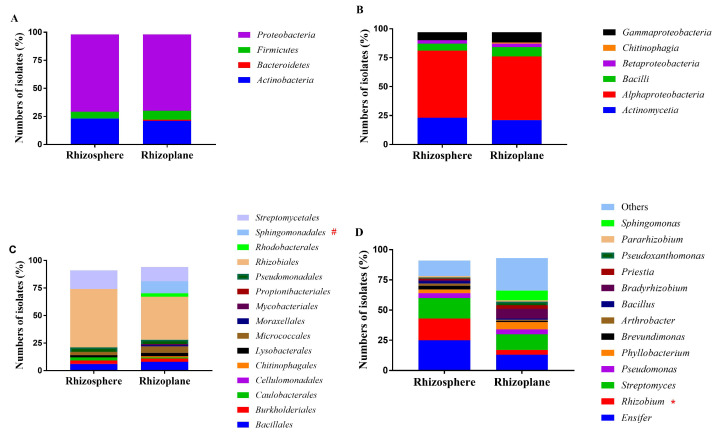
The taxonomic distribution of bacterial isolates, obtained from the rhizosphere and rhizoplane of *C. medica*, shown on Phylum (**A**), Class (**B**), Order (**C**), and Genera (**D**) level. Items labeled with * are taxa exhibiting significantly higher prevalence in the rhizosphere compared with the rhizoplane, while items with # are those with higher prevalence in the rhizoplane compared with the rhizosphere (Fisher’s exact test, *p* < 0.05).

In total, 174 bacterial isolates were obtained from the rhizosphere and rhizoplane of *C. medica* (61 from the rhizoplane and 113 from the rhizosphere). The 174 isolates belonged to four bacterial phyla: *Proteobacteria* (121 isolates), *Actinobacteria* (40), *Firmicutes* (12), and *Bacteroidetes* (1) (Figure 1 and Table S1). The four phyla were further divided into six classes. Three of these classes belonged to *Proteobacteria*: *Alpha-proteobacteria* (100 isolates, 56.9% of the total 174 isolates), *Gammaproteobacteria* (15 isolates, 8.6%), and *Beta-proteobacteria* (six isolates, 3.4%). The other three classes were *Actinomycetia* belonging to *Actinobacteria*, *Bacilli* belonging to *Firmicutes*, and *Chitinophagia* (one isolate) belonging to *Bacteroidetes*. The isolates were further classified into 15 orders, 24 families, and 38 genera. *Ensifer* (37 isolates), *Streptomyces* (28 isolates), and *Rhizobium* (24 isolates) were the most frequently identified genera (Figure 1 and Table S1).

### 3.2. Comparison of Taxonomic Distribution between Isolates from the Rhizoplane and Rhizosphere

Except for the sole isolate belonging to *Bacteroidetes*, which was obtained from the rhizoplane, the isolates belonging to the remaining three phyla had a similar prevalence in both the environments (Fisher’s exact test, *p* > 0.05). At the class level, the distribution of bacterial communities did not differ significantly between the rhizosphere and rhizoplane. However, the order *Sphingomonadales* was more prevalent in the rhizoplane (seven isolates, 12.96% of the total 61 isolates) than in the rhizosphere (one isolate, 0.89% of the total 113 isolates) (*p* < 0.05, Fisher’s exact test, two-tailed). In addition, isolates belonging to *Rhodobacterales* and *Chitinophagales* were only detected in the rhizoplane, while those belonging to *Propionibacteriales* and *Cellulomonadales* were only detected in the rhizosphere.

**Figure 2:**
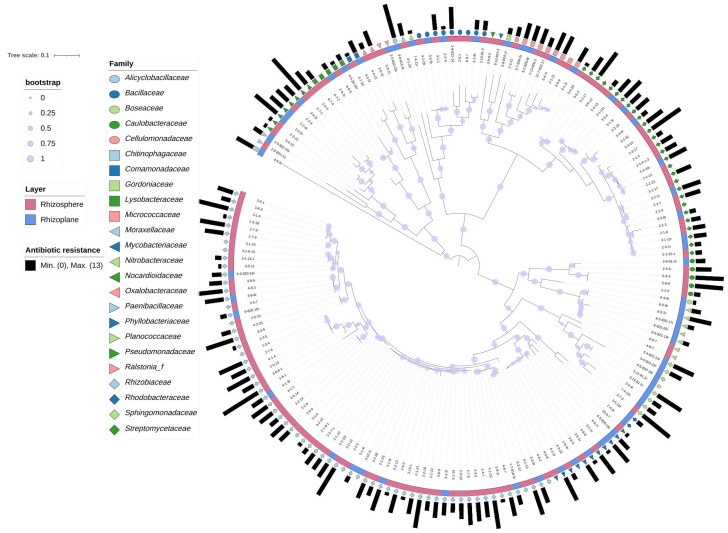
Phylogenetic tree, reconstructed based on the 16S rDNA sequences of the 174 isolates. The taxonomic information of the isolates is shown at the family level. The most inner circle is the isolate names. The second circle shows the origin of the isolates (rhizosphere—pink, rhizoplane—blue). The third circle is the taxonomic affiliation of the isolates. The outermost circle is the accumulated amount of antibiotics each isolate is resistant to.

At the family level, *Rhizobiaceae*, the predominant family, containing 54 isolates (47.79%) from the rhizosphere and 12 isolates (19.67%) from the rhizoplane, was less prevalent in the rhizoplane (*p* < 0.05, Fisher’s exact test, two-tailed) (Figure 2). Similar to the findings at the order level, the prevalence of isolates belonging to *Sphingomonadaceae*, a family belonging to *Sphingomonadales*, significantly differed between the rhizosphere and rhizoplane (*p* < 0.05, Fisher’s exact test, two-tailed). At the genus level, *Rhizobium* was found to be depleted in the rhizoplane [21 isolates (18.58%) from the rhizosphere and three isolates (4.92%) from rhizoplane] (*p* < 0.05, Fisher’s exact test, two-tailed). On the other hand, the prevalence of *Bradyrhizobium* was higher in the rhizoplane than in the rhizosphere [two isolates (1.77%) from the rhizosphere and five isolates (8.20%) from the rhizoplane]. A similar trend was noted in the prevalence of *Sphingomonas* (one isolate from rhizosphere and four isolates from rhizoplane), suggesting that these two genera were more abundant in the rhizoplane.

### 3.3. Comparison of Antibiotic Resistance Profiles between Isolates from the Rhizoplane and Rhizosphere

In total, 141 (81.03%) out of 174 isolates were found to be resistant to at least one of the 13 tested antibiotics [23] (Table S1). Several ARB, which are known to be opportunistic pathogens causing infectious diseases in clinical settings, were identified in this study. These included *Micrococcus luteus* (isolate 2-13-826-2), *Agrobacterium radiobacter* (isolate 2-8-1) [24], *Brevundimonas vesicularis* (isolates 2-5-6 and 3-8-91-3r) [25], and *B. diminuta* (isolates 2-2-9, 5-9-3 and 5-9-9) [26].

**Figure 3:**
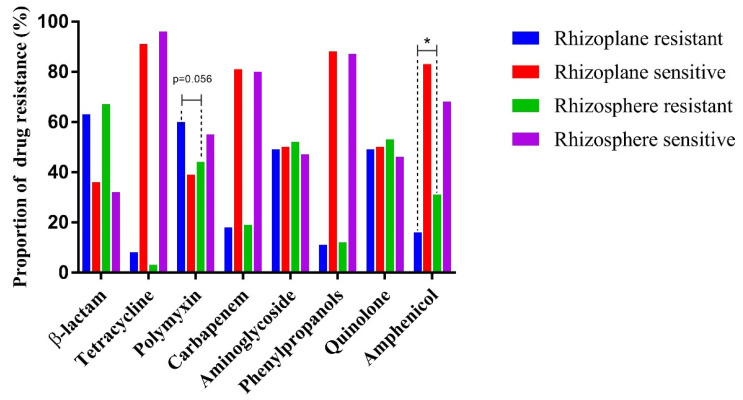
Antibiotic resistance profile differences between the rhizosphere and rhizoplane isolates. The prevalence difference of polymyxin and amphenicol-resistant isolates from the rhizosphere and rhizoplane was shown in the figure. Fisher exact test was used for antibiotic resistance profile comparison between the rhizosphere and rhizoplane isolates, and * denoted *p* < 0.05.

Most isolates from the rhizosphere and rhizoplane were sensitive to tetracycline (96.46% and 91.80%, respectively), phenylpropanol (87.61% and 88.52%, respectively), and carbapenems (80.53% and 81.97%, respectively) but resistant to β-lactam class antibiotics (67.26% and 63.93%, respectively) (Figure 3). The prevalence of isolates resistant to the aforementioned antibiotics did not differ significantly between the rhizosphere and rhizoplane (Fisher’s exact test, two-tailed, *p* > 0.05). However, the rhizoplane was found to harbor more antibiotic-resistant isolates (53 isolates, 86.89% of the total 61 isolates) than the rhizosphere (88 isolates, 77.88%), although the difference was not significant (Fisher’s exact test, two-tailed, *p* = 0.16).

Interestingly, the rhizoplane harbored more polymyxin-resistant isolates than the rhizosphere (60.66% and 44.25%, respectively) (Fisher’s exact test, two-tailed, *p* = 0.056). On the other hand, the rhizosphere harbored significantly higher numbers of amphenicol-resistant isolates than the rhizoplane (*p* < 0.05, Fisher’s exact test, two-tailed). The polymyxin-resistant isolates identified in the rhizoplane mainly belonged to 13 families, with *Rhizobiaceae* (20% of the total resistant isolates), *Streptomycetaceae* (20%), and *Sphingomonadaceae* (14%) being the predominant families (accounting for 60% of the identified polymyxin-resistant isolates). The polymyxin-resistant isolates identified in the rhizosphere mainly belonged to *Rhizobiaceae* and *Streptomycetaceae* (47% and 22% of the resistant isolates, respectively) (Table S1). Additionally, 7 (58%) out of 12 *Rhizobiaceae* isolates, 7 (88%) out of 8 *Streptomycetaceae* isolates, and 5 (71%) out of 7 *Sphingomonadaceae* isolates were resistant to polymyxin in the rhizoplane; however, 44% and 55% of the *Rhizobiaceae* and *Streptomycetaceae* isolates, respectively, were resistant to polymyxin in the rhizosphere. *Rhizobiaceae* also comprised the largest number of amphenicol-resistant isolates. The prevalence of amphenicol-resistant isolates in the rhizosphere was significantly higher than that in the rhizoplane (*p* < 0.05, Fisher’s exact test, two-tailed). Notably, we obtained seven *Bradyrhizobium* isolates; while four out of the five isolates from the rhizoplane were resistant to at least one antibiotic, both isolates from the rhizosphere were sensitive to all the tested antibiotics.

## 4. Discussion

In this study, 174 isolates were obtained from the root-associated niches of *C. medica*, including 113 isolates from the rhizosphere and 61 isolates from the rhizoplane. The prevalence of isolates belonging to certain taxa, such as *Sphingomonadales* and *Rhizobium*, significantly differed between the rhizosphere and rhizoplane. Moreover, several other taxa were detected only in the rhizosphere or rhizoplane, suggesting colonization and preference differences in the bacterial isolates located in the two distinct environments [13,27,28]. Several opportunistic pathogens, such as *M. luteus* isolate 2-13-826-2 and five *Brevundimonas* spp. isolates, were also detected, suggesting that the root-associated environments are important reservoirs of pathogens that can infect humans and may pose a serious threat to public health [9,10].

In this study, 81.03% (141 isolates) of the total 174 isolates were resistant to at least one antibiotic. The high prevalence of antibiotic-resistant isolates identified in this study might be, at least partially, due to the livestock manure applied to the trees, as well as the plant and microbiome-derived selective pressures for the bacteria colonizing the root-associated environments [13,27,28,29,30]. It is well-known that the livestock manures usually contain antibiotic residuals and ARB [29]. The antibiotic resistance and stress tolerance abilities of bacteria are also suggested to be positively correlated [30]. The root-associated bacteria, including that colonizing the rhizosphere and rhizoplane, play important roles in plant growth and health maintenance [13,14,15]. Several taxa that contain plant beneficial members, such as *Bradyrhizobium* spp. and *Variovorax* spp. [14,15], were also identified in this study. However, the antibiotic resistance of these root-associated bacteria could not be neglected. Our sampling sites were located in a park visited by tourists; thus, these sites were easily and frequently contacted by humans. The high antibiotic resistance of the isolates identified in our study illustrates the prevalence of ARB in the plant microbiome, which may threaten human health. Most isolates were resistant to β-lactam class antibiotics, including ampicillin, clofazimine, and cefotaxime. β-lactam class antibiotics contain a β-lactam ring in their chemical structure and are the most widely used antibiotics at present [31,32]. In recent years, β-lactam-resistant pathogens have become a serious threat in clinical settings [33]. Notably, the opportunistic pathogens identified in our study, including *M. luteus*, *A. radiobacter*, and *Brevundimonas* spp., were all resistant to at least one of the three tested β-lactam class antibiotics. Several infectious diseases caused by β-lactam-resistant *B. diminuta* have recently been reported to seriously impact human health [26]. The prevalence of polymyxin-resistant isolates was also very high in our study, accounting for 60.66% and 44.25% of the isolates from the rhizoplane and rhizosphere, respectively. Polymyxins have sometimes been the sole antimicrobial agents remaining active against pathogens that are resistant to other widely used antibiotics in clinical settings [34]. The emergence of polymyxin-resistant pathogens has caused several diseases and even deaths, posing a great threat to public health [35]. All the *M. luteus*, *A. radiobacter*, and *Brevundimonas* isolates (except for isolate 2-2-9) obtained in our study were resistant to polymyxin. These results suggest the potential of these ARB to infect humans and pose challenges to infection treatment and antibiotic usage. Moreover, our results highlight the importance of surveillance of these antibiotic-resistant opportunistic pathogens in environments that are closely contacted by humans. Compared with bacteria colonizing the rhizosphere, those colonizing the rhizoplane endure many fierce competitions from other microbes and face stronger selective pressures from the plant host, while also having easier access to plant-derived nutrients [13,14]. In this study, a higher percentage (86.89%) of isolates obtained from the rhizoplane exhibited resistance to at least one antibiotic than those obtained from the rhizosphere (77.88%). This result suggests that the antibiotic resistance could render bacteria more tolerant to environmental stresses [30]. Interestingly, more isolates from the rhizoplane (60.66%) than from the rhizosphere (44.25%) were resistant to polymyxin in our study (Fisher’s exact test, two-tailed, *p* = 0.056). On the other hand, the prevalence of chloramphenicol-resistant isolates was higher in the rhizosphere (31.86%) than in the rhizoplane (16.39%) (*p* < 0.05, Fisher’s exact test, two-tailed). These differences in antibiotic resistance profiles, between the bacterial isolates from the rhizosphere and rhizoplane, may be correlated with the selective pressure differences in the two environments and should be explored further in future research.

Overall, we obtained 174 bacterial isolates from the rhizosphere and rhizoplane of *C. medica* trees grown in a park, which humans could easily and frequently visit. The taxonomic that distribution of the isolates differed between the rhizosphere and rhizoplane, suggesting that nutrient availability and selective pressure differences between the two environments played an important role in shaping the bacterial communities colonizing the rhizosphere and rhizoplane. A very high proportion of isolates exhibited resistance to several clinically important antibiotics, including β-lactam class antibiotics and polymyxin, with several known antibiotic-resistant opportunistic pathogens being identified. However, the isolates were obtained from four trees in this study, so more comprehensive investigations on the ARB prevalence in the root-associated environments should be performed. Nevertheless, these results suggest both the risk of these pathogens in such closely related environments and the importance of surveillance of these antibiotic-resistant opportunistic pathogens in such environments. Furthermore, the prevalence of isolates resistant to certain antibiotics significantly differed between the two environments. These findings indicate the co-occurrence and, perhaps, co-evolution of antibiotic resistance and microbial and plant host-derived selective pressures in the two environments, which need to be assessed further in future research.

## Data Availability

The 16S rDNA sequences of the 174 bacterial isolates have been deposited in the NCBI GenBank database under accession numbers OP108637 to OP108810.

## Supplementary Materials

The following supporting information can be downloaded at: https://www.mdpi.com/article/10.3390/microorganisms10091708/s1, Table S1: Taxonomic distribution and antibiotic resistance profiles of the 174 bacterial isolates. Figure S1: The sample collection approach used for collecting the rhizosphere and rhizoplane soil samples of *C. medica* trees. The arrows denoted the four sites for sample collection.

## References

[1] Wang M., Tang J.C. (2010). Research of antibiotics pollution in soil environments and its ecological toxicity. J. Agro-Environ. Sci..

[2] Serwecińska L. (2020). Antimicrobials and Antibiotic-Resistant Bacteria: A Risk to the Environment and to Public Health. Water.

[3] Ondon B.S., Li S., Zhou Q., Li F. (2021). Sources of Antibiotic Resistant Bacteria (ARB) and Antibiotic Resistance Genes (ARGs) in the Soil: A Review of the Spreading Mechanism and Human Health Risks.

[4] Zhang Y.-J., Hu H.-W., Chen Q.-L., Singh B.K., Yan H., Chen D., He J.-Z. (2019). Transfer of antibiotic resistance from manure-amended soils to vegetable microbiomes. Environ. Int..

[5] Chen Q.-L., Cui H.-L., Su J.-Q., Penuelas J., Zhu Y.-G. (2019). Antibiotic Resistomes in Plant Microbiomes. Trends Plant Sci..

[6] Brandt K.K., Amézquita A., Backhaus T., Boxall A., Coors A., Heberer T., Lawrence J.R., Lazorchak J., Schönfeld J., Snape J.R. (2015). Ecotoxicological assessment of antibiotics: A call for improved consideration of microorganisms. Environ. Int..

[7] Huang R., Ding J., Guo Y., Sun B., Liang Y. (2022). Habitat determines the relationships among bacteria, resistance genes and mobile genetic elements in the soil–plant system. Eur. J. Soil Sci..

[8] Zhao W., Wang B., Yu G. (2018). Antibiotic resistance genes in China: Occurrence, risk, and correlation among different parameters. Environ. Sci. Pollut. Res. Int..

[9] Sharma R., Bisaria V.S., Sharma S. (2019). Rhizosphere: A Home for Human Pathogens.

[10] Berg G., Eberl L., Hartmann A. (2005). The rhizosphere as a reservoir for opportunistic human pathogenic bacteria. Environ. Microbiol..

[11] Cernava T., Erlacher A., Soh J., Sensen C.W., Grube M., Berg G. (2019). Enterobacteriaceae dominate the core microbiome and contribute to the resistome of arugula (*Eruca sativa* Mill.). Microbiome.

[12] Nygård K., Lassen J., Vold L., Andersson Y., Fisher I., Löfdahl S., Threlfall J., Luzzi I., Peters T., Hampton M. (2008). Outbreak of *Salmonella* Thompson infections linked to imported rucola lettuce. Foodborne Pathog. Dis..

[13] Reinhold-Hurek B., Bünger W., Burbano C.S., Sabale M., Hurek T. (2015). Roots Shaping Their Microbiome: Global Hotspots for Microbial Activity. Annu. Rev. Phytopathol..

[14] Zhang Y., Xu J., Riera N., Jin T., Li J., Wang N. (2017). Huanglongbing impairs the rhizosphere-to-rhizoplane enrichment process of the citrus root-associated microbiome. Microbiome.

[15] Zhang Y., Trivedi P., Xu J., Roper M.C., Wang N. (2021). The Citrus Microbiome: From Structure and Function to Microbiome Engineering and Beyond. Phytobiomes J..

[16] Kawasaki K., Kamagata Y. (2017). Phosphate-Catalyzed Hydrogen Peroxide Formation from Agar, Gellan, and kappa-Carrageenan and Recovery of Microbial Cultivability via Catalase and Pyruvate. Appl. Environ. Microbiol..

[17] Kato S., Yamagishi A., Daimon S., Kawasaki K., Tamaki H., Kitagawa W., Abe A., Tanaka M., Sone T., Asano K. (2018). Isolation of Previously Uncultured Slow-Growing Bacteria by Using a Simple Modification in the Preparation of Agar Media. Appl. Environ. Microbiol..

[18] Yoon S.-H., Ha S.-M., Kwon S., Lim J., Kim Y., Seo H., Chun J. (2017). Introducing EzBioCloud: A taxonomically united database of 16S rRNA gene sequences and whole-genome assemblies. Int. J. Syst. Evol. Microbiol..

[19] Nguyen L.-T., Schmidt H.A., Von Haeseler A., Minh B.Q. (2015). IQ-TREE: A fast and effective stochastic algorithm for estimating maximum-likelihood phylogenies. Mol. Biol. Evol..

[20] Letunic I., Bork P. (2021). Interactive Tree Of Life (iTOL) v5: An online tool for phylogenetic tree display and annotation. Nucleic Acids Res..

[21] Wiegand I., Hilpert K., Hancock R.E.W. (2008). Agar and broth dilution methods to determine the minimal inhibitory concentration (MIC) of antimicrobial substances. Nat. Protoc..

[22] European Committee for Antimicrobial Susceptibility Testing (EUCAST) of the European Society of Clinical Microbiology and Infectious Diseases (ESCMID) (2003). Determination of minimum inhibitory concentrations (MICs) of antibacterial agents by broth dilution. Clin. Microbiol. Infect..

[23] Hutchings M.I., Truman A.W., Wilkinson B. (2019). Antibiotics: Past, present and future. Curr. Opin. Microbiol..

[24] Szczesny G., Leszczynski P., Sokol-Leszczynska B., Maldyk P. (2022). Identification of human-dependent routes of pathogen’s transmission in a tertiary care hospital. Jt. Dis. Relat. Surg..

[25] Balmer L., Seth-Smith H.M.B., Egli A., Casanova C., Kronenberg A., Schrenzel J., Marschall J., Sommerstein R. (2022). Agrobacterium species bacteraemia, Switzerland, 2008 to 2019: A molecular epidemiological study. Antimicrob. Resist. Infect. Control.

[26] Ryan M.P., Pembroke J.T. (2018). *Brevundimonas* spp: Emerging global opportunistic pathogens. Virulence.

[27] Beckers B., Op De Beeck M., Weyens N., Boerjan W., Vangronsveld J. (2017). Structural variability and niche differentiation in the rhizosphere and endosphere bacterial microbiome of field-grown poplar trees. Microbiome.

[28] Zhang Y., Xu J., Wang E., Wang N. (2020). Mechanisms Underlying the Rhizosphere-To-Rhizoplane Enrichment of *Cellvibrio* Unveiled by Genome-Centric Metagenomics and Metatranscriptomics. Microorganisms.

[29] Xie W., Shen Q., Zhao F.J. (2018). Antibiotics and antibiotic resistance from animal manures to soil: A review. Eur. J. Soil Sci..

[30] Liao X., Ma Y., Daliri E.B.-M., Koseki S., Wei S., Liu D., Ye X., Chen S., Ding T. (2020). Interplay of antibiotic resistance and food-associated stress tolerance in foodborne pathogens. Trends Food Sci. Technol..

[31] Carvalho I.T., Santos L. (2016). Antibiotics in the aquatic environments: A review of the European scenario. Environ. Int..

[32] Bush K., Bradford P.A. (2020). Bradford, Epidemiology of β-Lactamase-Producing Pathogens. Clin. Microbiol. Rev..

[33] Ngoi S.T., Muhamad A.N., The C.S.J., Chong C.W., Abdul Jabar K., Chai L.C., Leong K.C., Tee L.H., AbuBakar S. (2021). beta-Lactam Resistance in Upper Respiratory Tract Pathogens Isolated from a Tertiary Hospital in Malaysia. Pathogens.

[34] Nang S.C., Azad M.A.K., Velkov T., Zhou Q., Li J. (2021). Rescuing the Last-Line Polymyxins: Achievements and Challenges. Pharmacol. Rev..

[35] Liu B.-T., Li X., Zhang Q., Shan H., Zou M., Song F.-J. (2019). Colistin-Resistant mcr-Positive Enterobacteriaceae in Fresh Vegetables, an Increasing Infectious Threat in China. Int. J. Antimicrob. Agents.

