# Supplementary material for: High Prevalence and Varied Distribution of Antibiotic-Resistant Bacteria in the Rhizosphere and Rhizoplane of Citrus medica
Source: Microorganisms. 2022 Aug 25;10(9):1708. doi: 10.3390/microorganisms10091708 (PMC9501533; doi:10.3390/microorganisms10091708)
Supplement: Supplementary file 1 [file microorganisms-10-01708-s001.zip › Figure S1..pdf]

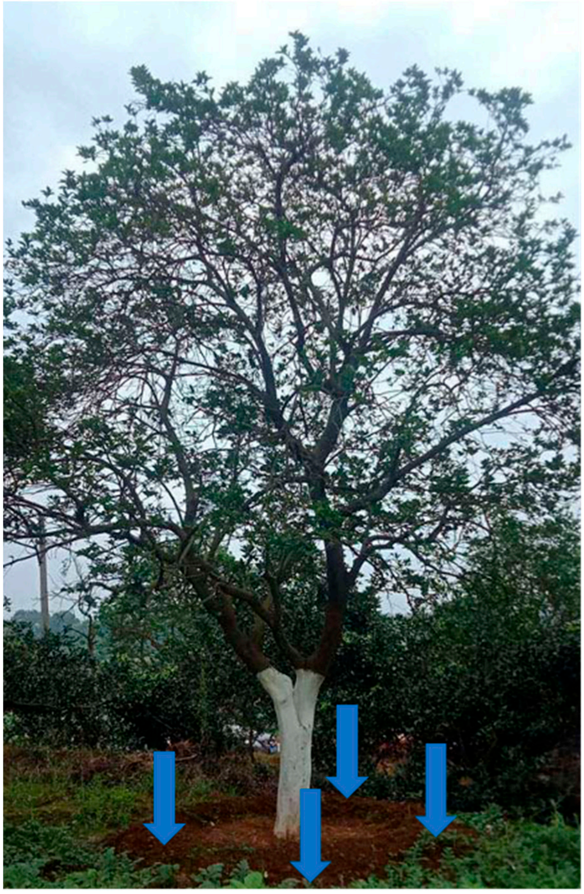

Figure S1. The sample collection approach used for collecting the rhizosphere and rhizoplane soil samples of *C. medica* trees. The arrows denoted the four sites for sample collection.
